# Pedicle Flap Combined With Soft Tissue Graft for the Management of an Isolated Deep Buccal Recession in Auto-Transplanted Maxillary Molar: A Case Report

**DOI:** 10.7759/cureus.76880

**Published:** 2025-01-03

**Authors:** Muataz Banjar, Amal S Alqahtani, Raghad H Alsulami

**Affiliations:** 1 Periodontics and Implant Dentistry, King Faisal Hospital, Makkah, SAU; 2 Dentistry, Umm Al-Qura University, Makkah, SAU

**Keywords:** auto-transplanted tooth, case report, gingival recession, keratinized tissue, mucogingival, soft tissue graft

## Abstract

This case report introduced a new technique to manage buccal deep isolated recession with loss of keratinized tissue. This technique is indicated where there is a good amount of keratinized tissue lateral to the defect and deep vestibule. In this case report, a 27-year-old male patient was referred to a periodontal surgery clinic for the management of an 8 mm buccal recession with loss of keratinized tissue related to auto-transplanted maxillary molar that caused discomfort to the patient. The denuded root is covered by a laterally and coronally advanced flap combined with a soft tissue graft using only one vertical incision. This technique showed good results in gaining keratinized tissue with partial coverage of the root for the auto-transplanted maxillary molar. Further studies are recommended for the management of periodontally involved auto-transplanted teeth.

## Introduction

Gingival recession is known as the apical movement of the gingival margin relative to the cemento-enamel junction (CEJ) of the tooth [1]. Many factors contribute to the development of gingival recession, such as the absence of attached gingiva, reduced alveolar bone thickness, and malposition of the tooth in the dental arch. The presence of attached gingiva is of paramount importance in maintaining gingival health [2]. Cortellini and Bissada suggested that about 2 mm of keratinized tissue (KT) and about 1 mm of attached gingiva are essential around the tooth to maintain periodontal health. However, meticulous oral hygiene could prevent the disease even in the presence of a minimal amount of KT [3]. A cross-sectional study was conducted in France to identify variables related to buccal gingival recessions in adults, and it was found that around 85% of the sample had at least one gingival recession [4]. However, based on our search, no studies have been conducted on the prevalence of gingival recession in the Saudi population.

Dentin hypersensitivity, esthetic problems, and the increased risk of destruction of the tooth through root caries and non-carious cervical lesions (NCCLs) are consequences that may occur due to root surface exposure to the oral environment [3].

Management of gingival recession, either to change the gingival phenotype and/or cover the root, may be indicated if the risk of recession progression and associated root damage is present or if oral hygiene measures cannot be adequately maintained [3]. Several surgical techniques have been reported for the treatment of gingival recession, including the use of epithelized free gingival grafts or connective tissue grafts (CTGs) with varied flap designs (i.e., envelope, double pedicle flap, tunnel, laterally or coronally positioned flap, and laterally closed or combination of them) [5-10]. Grupe and Warren first proposed the laterally positioned flap technique to utilize the KT of a tooth adjacent to a gingival recession that is repositioned laterally to cover the exposed root [11]. Our suggested technique builds on the historical surgical technique with the addition of an epithelized free gingival graft; half of it remains epithelized, and the other half is prepared as CTG for root surface coverage. In consideration of this, the given surgical approach may provide an effective technique to manage deep isolated recession.

## Case presentation

**Figure 1:**
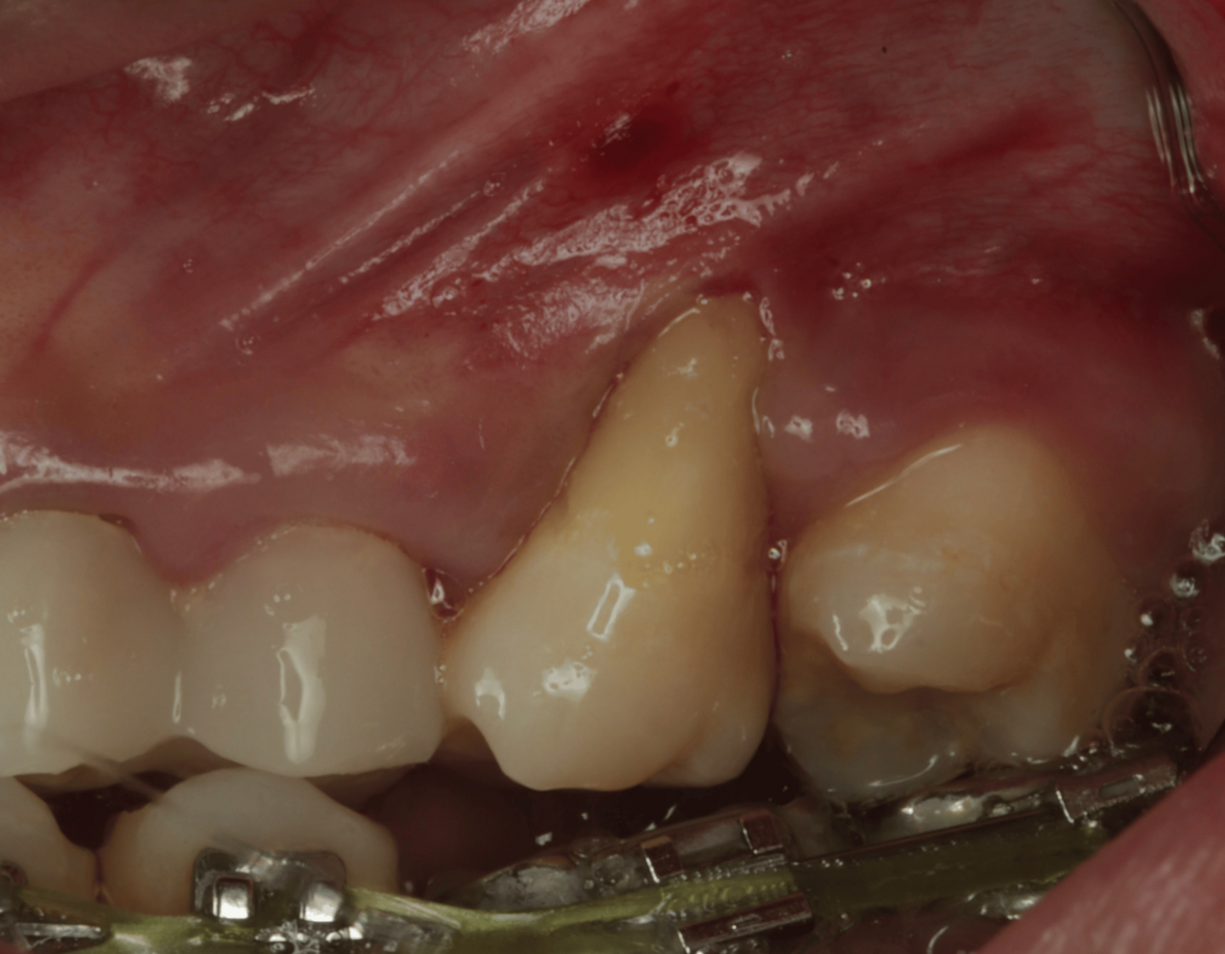
Initial situation, presented with isolated deep buccal recession 8 mm.

This case report is based on the case report guidelines [12]. In November 2024, at King Faisal Hospital in Makkah, Saudi Arabia. A 27-year-old systematically healthy male was referred from the endodontic clinic after the auto-transplantation of tooth #28 to replace non-restorable tooth #26. The clinical examination evidenced a single isolated deep recession 8 mm depth, pocket depth = 1 mm buccally, and clinical attachment loss buccally = 9mm, without bleeding on probing nor clinical attachment loss at interproximal attachment, with the absence of KT apically. The tooth developed Cairo type 1 (RT-1) gingival recession with a minimal amount of KT, which led to discomfort during brushing. There was also a wide band of KT adjacent to the recession 4 mm and a deep vestibular depth (Figure 1).

**Figure 2:**
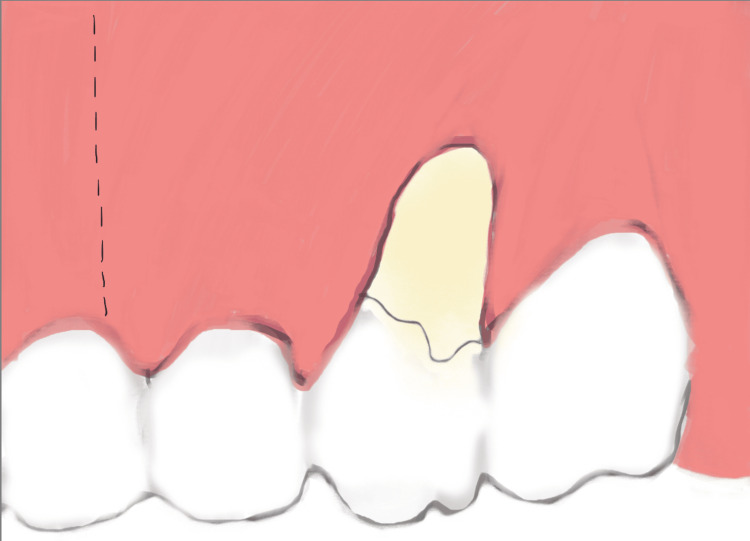
Vertical incision.

**Figure 3:**
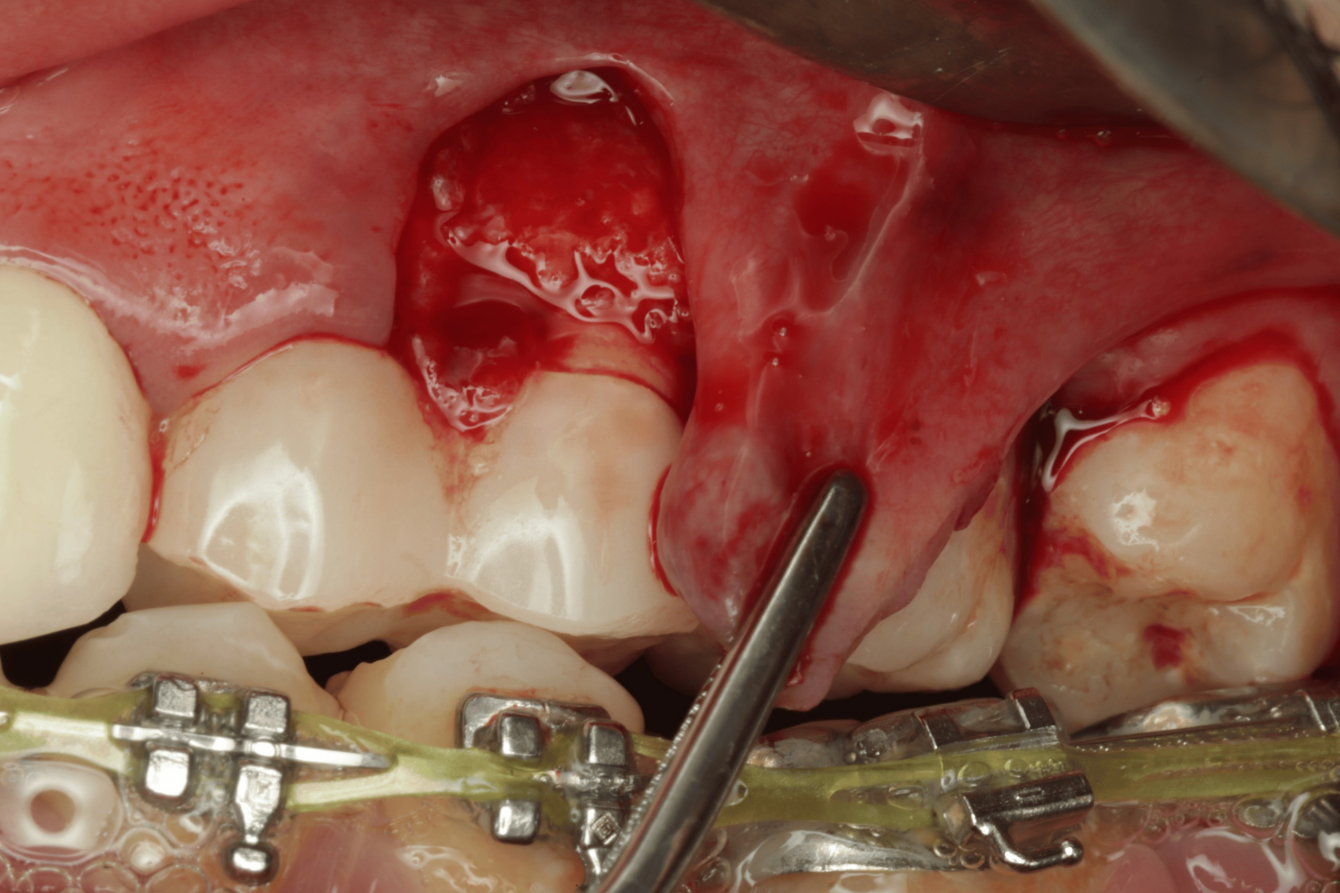
The amount of the flap released.

**Figure 4:**
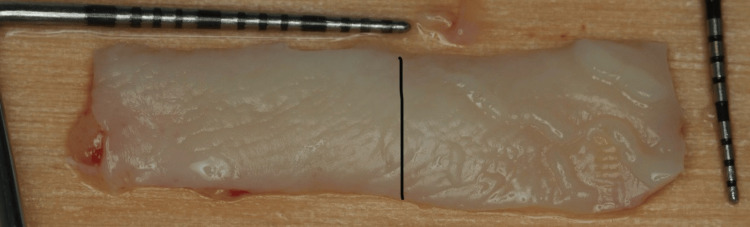
The harvested free gingival graft, half of it epithilized and the other half will be de-epithlized.

**Figure 5:**
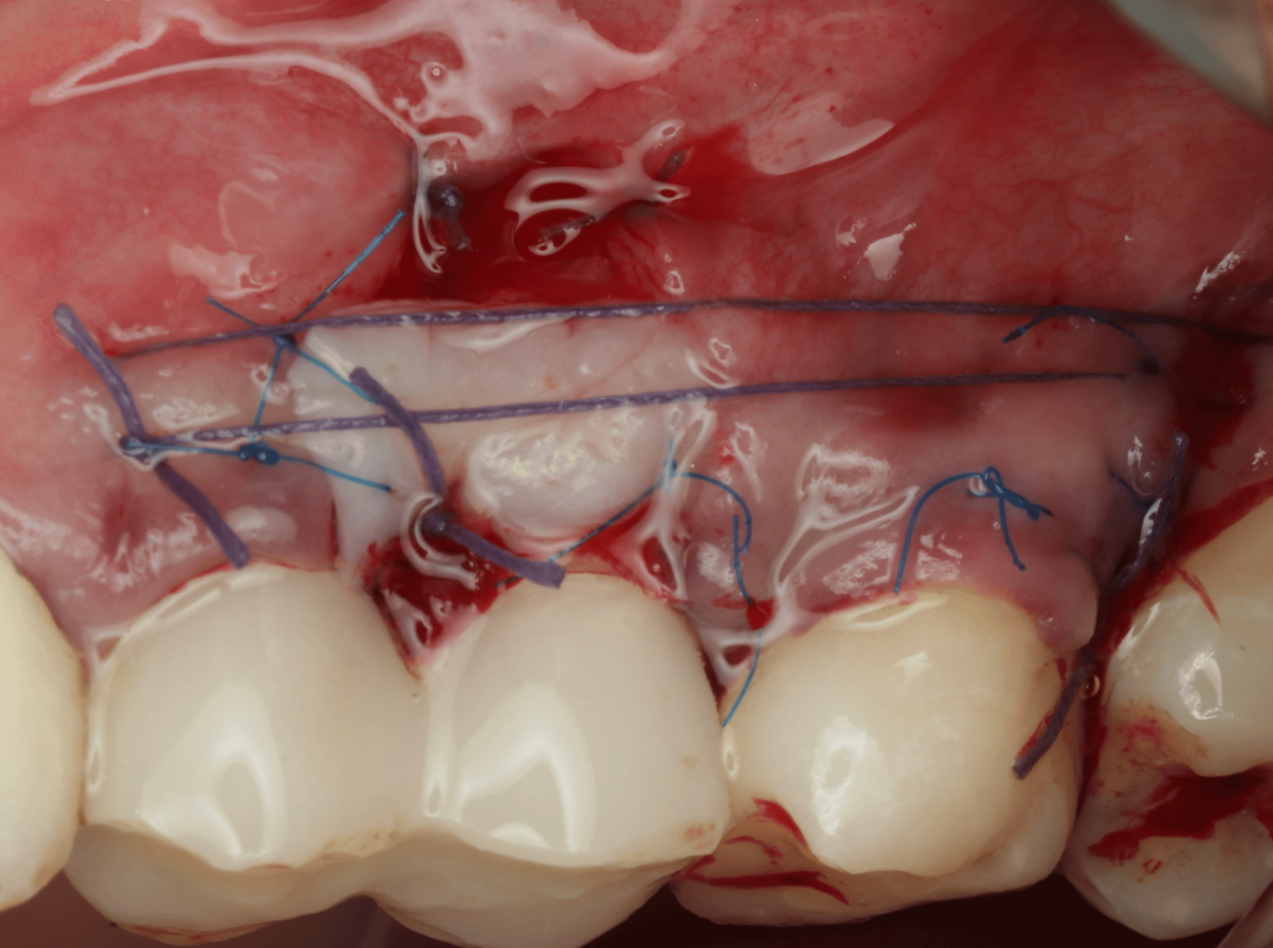
Soft tissue graft fixation with simple interrupted suture, horizontal suture, and sling suture.

**Figure 6:**
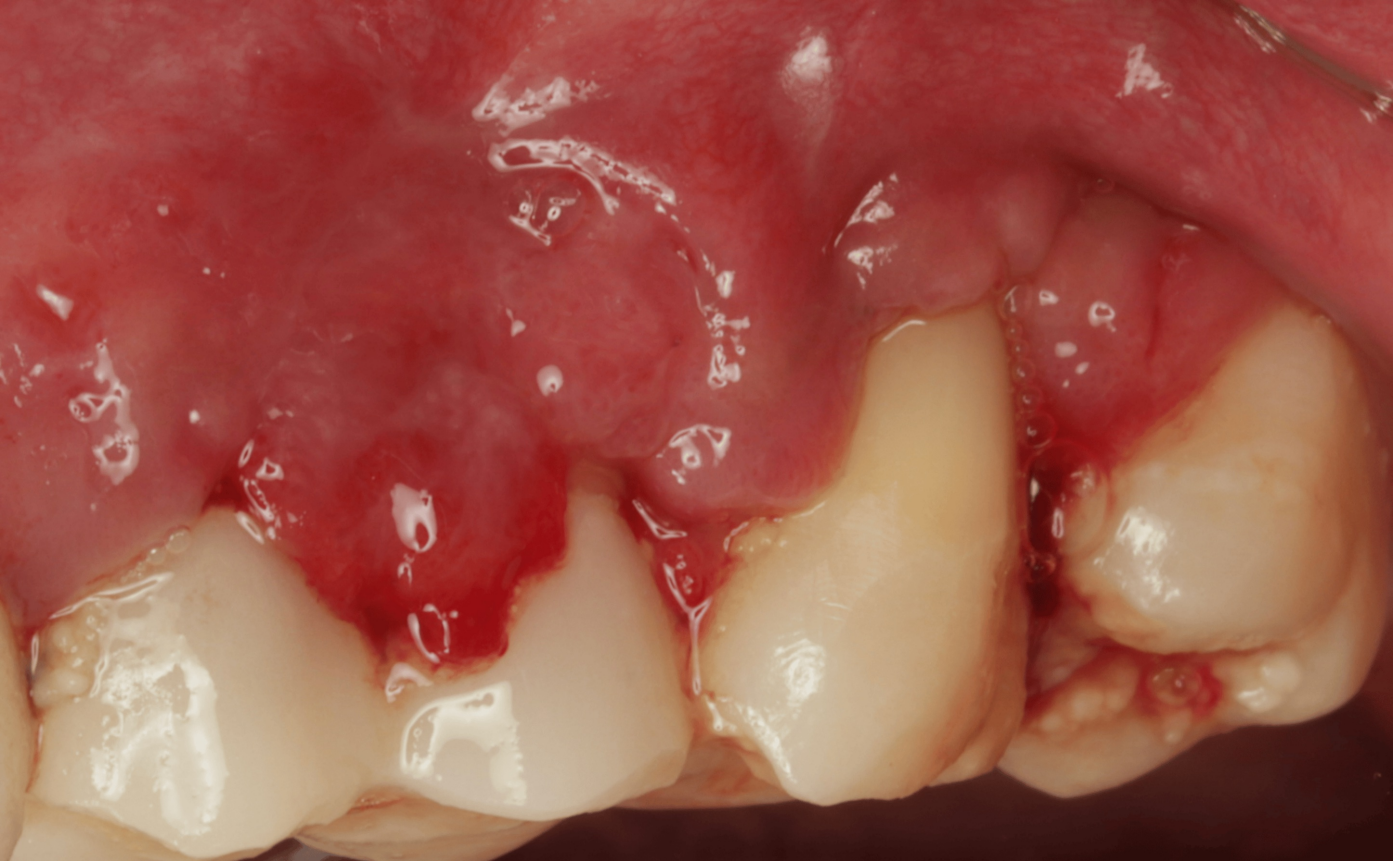
Two-week follow-up after removing the suture. The tissue is still edematous.

Two weeks before the surgery, the patient was instructed to maintain good oral hygiene by using a soft dental brush and following the modified Stillman technique with dental flossing, and supra-gingival prophylaxis was performed using an ultrasonic scaler and polishing paste. Before starting the surgery, written informed consent was obtained from the patient. After delivering local infiltration anesthesia (Articaine hydrochloride 4% with 1/100000 epinephrine), the surgical procedure was conducted as follows: a vertical incision using no. 15C blade starting at the distal line angle of tooth #24 and extending deep into the alveolar mucosa (Figure 2) and reflecting a partial thickness flap above the mucogingival line to allow sufficient flap release and improve blood supply (Figure 3). The denuded root surface of tooth #26 was gently prepared with fine diamond bur and Gracey curette 9-10 (HuFriedyGroup, Chicago, IL) to remove any irregularities, flattening the root surface and removing the smear layer. Infiltration local anesthesia (Articaine hydrochloride 4% with 1/100000 epinephrine) was given at the upper left lateral side of the palate to harvest free gingival graft using no. 15 blade. The graft was harvested from the palatal left side, and the first horizontal incision was established 3 mm away from the palatal gingival margin. A vertical incision was made medial to tooth #24 and extended distally to tooth #26. The size of the graft (8 mm width × 24 mm length), half of the graft is left as epithelized free gingival graft, and the other half of the graft was de-epithelized extra-orally using no. 15 blade (Figure 4). The graft was then placed on the prepared site; the epithelized part covered the recipient site tooth #25, and the de-epithelized connective tissue part covered the recession defect tooth #26. Simple interrupted using 5-0 (Polyglactin 910 absorbable surgical suture, UniMed Medical, Shenzhen, China) suture to stabilize the epithelized free gingival graft on tooth #25. Then, the released flap is positioned in a lateral and more coronal position to cover the CTG on tooth #26 with a sling suture using a 5-0 (Polyglactin 910 absorbable surgical suture) suture (Figure 5). The palatal donor site was left uncovered and healed by secondary intention. The patient received instructions to adhere to a soft diet and to rinse with chlorhexidine 0.12% twice a day for 14 days. As for anti-inflammatory and analgesic medications, ibuprofen 400 mg (3×/day) were prescribed. After 14 days, the sutures were removed (Figure 6).

After 14 days, the sutures were removed (Figure 5). 

**Figure 7:**
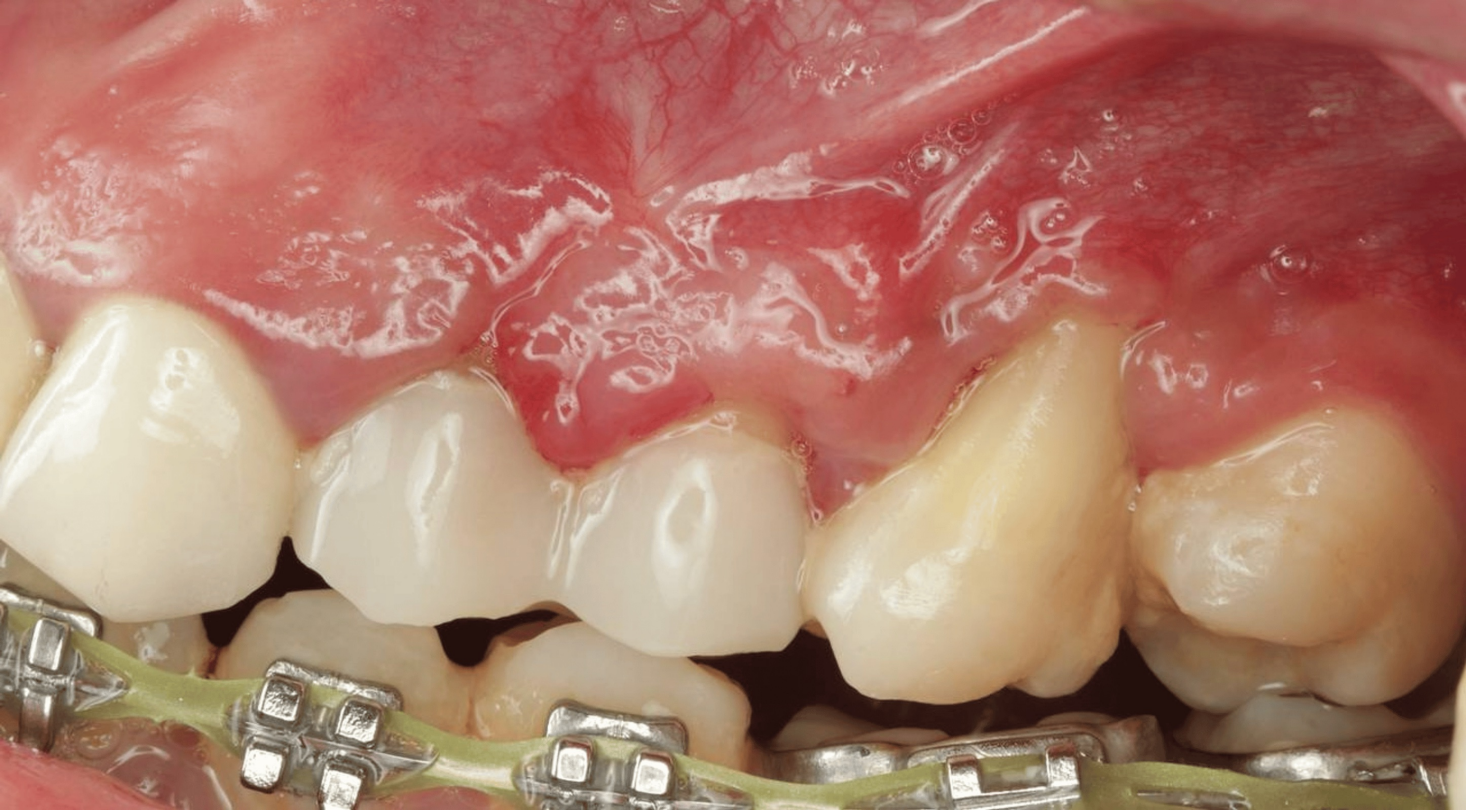
Six-week follow-up. The keratinized tissue was significantly increased.

At the six-week follow-up, the patient presented with a good amount of KT, which gained about 3 mm. Although complete root coverage was not expected, about 25% of the denuded root was covered on tooth #26, and the gingival thickness was notably increased (Figure 7).

## Discussion

The clinical case illustrated a technique for the management of deep recession at auto-transplanted tooth #26, which was buccally prominent, out of the bony housing, with loss of KT. Even though other surgical techniques could be suitable for this critical clinical scenario, it is important to assess all the key elements that lead our suggested surgical technique to be clinically successful, as shown in gaining KT, obtaining good root coverage, and increasing soft tissue volume. 

Gaining KT is of paramount importance since it acts as a protective barrier that resists mechanical trauma from brushing and mastication. Also, it maintains effective oral hygiene and provides better functional outcomes by supporting the natural contour of the gingiva [13].

One of the necessary components of the proposed method is the existence of a band of KT lateral to the recession defect that permits covering the exposed root surface. Despite the fact that most of the previous clinical studies have not found the minimum thickness and height of KT lateral to gingival defects needed to provide the laterally moved flap a predictable root coverage procedure, a particular height of KT should be preserved to reduce the risk of gingival recession at donor site [14-16].

Furthermore, another significant surgical factor is the elevation of a split-thickness flap, which provides flap passivity and displacement. Notably, the only vertical incision, which is the initial split-thickness incision, created a recipient bed for the CTG. Additionally, donor site #25 received the epithelized portion of the graft to reduce morbidity at the donor site.

Root planing and cementoplasty play important roles in achieving successful management outcomes. Soft tissue grafting contributes to a considerable increase in tissue volume and the gain of keratinized mucosa, thereby enhancing the predictability of the root coverage procedure. It is important to note that incomplete root coverage can result when the tooth is positioned outside its bony housing. Given the morphological and anatomical differences between tooth #28 and tooth #26, it was evident that the root of tooth #28 was prominent and had been auto-transplanted into a socket with a completely lost buccal plate. This characteristic reduces the predictability of root coverage.

After establishing a sufficient amount of KT, performing a coronally advanced flap is recommended to achieve greater coverage of the root surface.

## Conclusions

This case report is a valuable insight into the management of complications associated with auto-transplanted teeth. The presented technique is a predictable procedure for treating loss of KT in the isolated deep buccal recession in cases where the KT adjacent to the gingival defects is available. Further future studies are recommended regarding the complications related to the periodontal tissue around the auto-transplanted teeth.

## References

[1] Pini Prato G (1999). Mucogingival deformities. Ann Periodontol.

[2] Kim DM, Neiva R (2015). Periodontal soft tissue non-root coverage procedures: a systematic review from the AAP Regeneration Workshop. J Periodontol.

[3] Cortellini P, Bissada NF (2018). Mucogingival conditions in the natural dentition: narrative review, case definitions, and diagnostic considerations. J Periodontol.

[4] Sarfati A, Bourgeois D, Katsahian S, Mora F, Bouchard P (2010). Risk assessment for buccal gingival recession defects in an adult population. J Periodontol.

[5] Sullivan HC, Atkins JH (1968). Free autogenous gingival grafts. I. Principles of successful grafting. Periodontics.

[6] Sculean A, Allen EP (2018). The laterally closed tunnel for the treatment of deep isolated mandibular recessions: surgical technique and a report of 24 cases. Int J Periodontics Restorative Dent.

[7] Tunkel J, Hofmann F, de Stavola L (2021). The multiple pedicle coronally advanced flap for multiple deep Miller Class II recessions: a case report. Clin Adv Periodontics.

[8] Zabalegui I, Sicilia A, Cambra J, Gil J, Sanz M (1999). Treatment of multiple adjacent gingival recessions with the tunnel subepithelial connective tissue graft: a clinical report. Int J Periodontics Restorative Dent.

[9] Allen AL (1994). Use of the supraperiosteal envelope in soft tissue grafting for root coverage. I. Rationale and technique. Int J Periodontics Restorative Dent.

[10] Zucchelli G, Cesari C, Amore C, Montebugnoli L, De Sanctis M (2004). Laterally moved, coronally advanced flap: a modified surgical approach for isolated recession-type defects. J Periodontol.

[11] Grupe HE, Warren Jr RF (1956). Repair of gingival defects by a sliding flap operation. J Periodontol.

[12] Gagnier JJ, Kienle G, Altman DG, Moher D, Sox H, Riley D (2013). The CARE guidelines: consensus-based clinical case reporting guideline development. Headache.

[13] Lang NP, Löe H (1972). The relationship between the width of keratinized gingiva and gingival health. J Periodontol.

[14] Espinel MC, Caffesse RG (1981). Comparison of the results obtained with the laterally positioned pedicle sliding flap-revised technique and the lateral sliding flap with a free gingival graft technique in the treatment of localized gingival recessions. Int J Periodontics Restorative Dent.

[15] Guinard EA, Caffesse RG (1978). Treatment of localized gingival recessions. Part III. Comparison of results obtained with lateral sliding and coronally repositioned flaps. J Periodontol.

[16] Smukler H (1976). Laterally positioned mucoperiosteal pedicle grafts in the treatment of denuded roots. A clinical and statistical study. J Periodontol.

